# Streamlined Management of Basal Cell Carcinoma with Dermoscopy: A Retrospective Case–Control Study

**DOI:** 10.3390/jcm14248945

**Published:** 2025-12-18

**Authors:** Francisca Donoso, Rosario Aguero, Marie-Chantal Caussade, Dominga Peirano, Leonel Hidalgo, Sofía Villagrán, Pascal De Amesti, Víctor Meza, Josefina Hasenberg, Katherine Droppelmann, Álvaro Abarzúa-Araya, Juan Camilo Castro-Ayala, John Paoli, Pablo Uribe, Cristián Navarrete-Dechent

**Affiliations:** 1 Department of Dermatology, Escuela de Medicina, Pontificia Universidad Católica de Chile, Santiago 8330077, Chile; fdonoso4@uc.cl (F.D.); raguerou@gmail.com (R.A.); mcaussade@miuandes.cl (M.-C.C.); leonelhidalgo@gmail.com (L.H.); spvillagran@uc.cl (S.V.); kathydroppelmann@gmail.com (K.D.); alvaroabarzuaaraya@gmail.com (Á.A.-A.); juancamilocastroayala@gmail.com (J.C.C.-A.); 2 Department of Dermatology, Escuela de Medicina, Universidad de los Andes, Santiago 7620086, Chile; dominga.peirano@gmail.com (D.P.);; 3 Melanoma and Skin Cancer Unit, Escuela de Medicina, Pontificia Universidad Católica de Chile, Santiago 7620086, Chile; 4 Department of Dermatology and Venereology, Region Västra Götaland, Sahlgrenska University Hospital, 413 45 Gothenburg, Sweden; 5 Department of Dermatology and Venereology, Institute of Clinical Sciences, Sahlgrenska Academy, University of Gothenburg, 405 30 Gothenburg, Sweden

**Keywords:** basal cell carcinoma, surgery, skin cancer, wide local excision, Mohs micrographic surgery

## Abstract

**Background/Objective:** The standard approach for managing suspected basal cell carcinoma (BCC) involves performing a biopsy to confirm the diagnosis before treatment. This process often leads to multiple visits and increased healthcare costs. We aimed to evaluate the effectiveness of direct surgical excision of BCCs diagnosed clinically and dermoscopically, without the need for prior biopsy. **Methods:** We conducted a retrospective case–control study at a tertiary cancer center. Lesions suspected to be BCC, based on clinical and dermoscopic criteria, were divided into two groups: (1) a streamlined treatment group (cases), in which lesions were treated without a confirmatory biopsy (either excised with a 4 mm margin or managed with curettage and electrodesiccation); (2) and a biopsied group (controls). Clinical and histopathological data were analyzed and compared between groups to assess diagnostic accuracy, margin status, and treatment outcomes. **Results:** Of 389 BCCs, 167 (42.9%) were streamlined, while 222 (57.1%) underwent a biopsy before definitive treatment. The streamlined group demonstrated higher diagnostic accuracy, with 94.6% of excised lesions confirmed as BCC, compared with 73.4% in the biopsy group (*p* < 0.001). Among lesions excised with 4 mm margins, 97.9% achieved clear margins with the streamlined approach. Margin involvement was associated with high-risk BCC (*p* = 0.048), particularly with recurrent BCCs (*p* = 0.023). **Conclusions:** Streamlined management of BCC through direct excision without prior biopsy is an efficient and cost-effective strategy that reduces patient visits, costs, and waiting times, particularly for low-risk BCCs and older patients. Advances in dermoscopy and non-invasive tools support their accuracy, making it a feasible option in resource-limited settings.

## 1. Introduction

The gold standard for treating basal cell carcinoma (BCC) mandates conducting a biopsy before definitive treatment, as emphasized by both the National Comprehensive Cancer Network and the American Academy of Dermatology guidelines [1,2]. This protocol often requires multiple visits, resulting in extended waiting times and escalating costs for patients and the healthcare system. However, with the increasing adoption of dermoscopy and other recent non-invasive techniques, such as reflectance confocal microscopy (RCM), and optical coherence tomography (OCT), clinicians now possess powerful diagnostic tools that can significantly enhance the accuracy of their initial clinical assessments [3,4].

Some authors have proposed a more ‘streamlined’ method, where the tumor is excised without a preceding biopsy. Studies have shown that this strategy is not only cost-efficient but also maintains the quality of care when compared with conventional management [5,6]. This streamlined approach, which involves removing the lesion directly with the intention to treat (and not only to ‘sample’) based on clinical and dermoscopic diagnosis, minimizes the number of required visits and can expedite the initiation of definitive treatment [7].

This strategy has been used routinely in our center for lesions highly suggestive of BCC under dermoscopy, with careful evaluation, on a case-by-case basis. Each patient’s characteristics, the associated risks of BCC, and treatment options are considered to ensure an individualized approach. The primary objective of this study was to assess the effectiveness of streamlined management for BCCs identified through clinical and dermoscopic examination. By comparing outcomes with those of traditional management, we aim to highlight the potential benefits of this streamlined approach to patient care, eliminating the need for a prior biopsy.

## 2. Patients and Methods

### 2.1. Study Design

A retrospective case–control study was conducted at a tertiary cancer center in Santiago, Chile (IRB# 211213001) reviewing the consecutive biopsy reports from cases submitted with the differential diagnosis of BCC obtained by 6 dermatologists at a skin cancer unit from April 2021 to January 2022. All 6 dermatologists had formal training in dermoscopy and a minimum of ten years of experience in the clinical and dermoscopic diagnosis of BCC. Lesions were allocated to management strategies based on clinical judgment rather than formal matching. The streamlined group included lesions considered suitable for direct removal without prior biopsy, based on standardized clinical and dermoscopic features commonly associated with BCC, such as arborizing vessels, shiny white structures, leaf-like areas, spoke-wheel structures, and blue-grey ovoid nests, as assessed by the treating dermatologist and described in the systematic review by Reiter et al., 2019 [3]. The biopsy group included lesions that underwent biopsy prior to management. Decisions were made according to clinician preference, dermoscopic confidence, lesion characteristics, and patient factors. Only cases that had a biopsy performed (control group) and those with streamlined management with curative intent without prior biopsy (streamlined group), i.e., either by excision with 4 mm clinical margins or via curettage and electrodesiccation (C&ED), were included. Cases that were treated with Mohs micrographic surgery (MMS) or BCCs undergoing re-excision due to a previous incomplete excision were excluded.

For all lesions in the streamlined group, a final histopathological diagnosis was obtained. For the 4 mm excision group, tissue was sent for histopathological examination and postoperative margin assessment as per NCCN guidelines [1]. Postoperative histopathological evaluation and margin assessment were performed using the standard bread-loafing technique. The definition of a negative margin was standardized across all pathology reports. For lesions treated with C&ED, a broad shave biopsy was performed prior to the procedure and sent for postoperative histopathological evaluation.

### 2.2. Data Collection

Data was extracted from electronic medical records and histopathology reports. The following variables were described for both groups: age, sex, location by anatomical zone (H, high risk; M, medium risk, and L, low risk) [8], and specific anatomical site (i.e., head, neck, chest, abdomen, back, anogenital area, upper extremities, and lower extremities). Risk classification for BCCs was performed according to NCCN guidelines [1], which define high-risk lesions based on criteria such as anatomical location in high-risk areas (i.e., H zone), aggressive histopathological subtype (morpheaform, infiltrative, micronodular, basosquamous), recurrence, perineural invasion, prior radiotherapy, or history of immunosuppression. These are some of the main criteria, and all of these variables were also documented for each case. The final histopathological diagnosis (BCC or other), and histopathological margin involvement in streamlined excisions were also recorded.

### 2.3. Statistical Analysis

Quantitative variables were described using means and standard deviations, while qualitative or categorical variables were expressed as frequencies and percentages. The Pearson Chi-square test was applied for associations between categorical variables, and the student’s *t*-test was used for continuous variables associated with two-level categorical variables. When the expected frequency for a combination of variables was less than 5, Fisher’s exact test was performed. To identify independent predictors of management strategy (streamlined vs. biopsy), a multivariable logistic regression model was performed, including patient age, anatomical location (H, M, L zones), and aggressive histopathological subtype as covariates. A 95% confidence interval was reported for all significant findings. The number of cases with margin involvement or diagnostic discordance was too small to support a reliable multivariable analysis for these outcomes. A *p*-value < 0.05 was considered statistically significant. Statistical analyses were performed using SPSS software version 26.0 (IBM, Armonk, NY, USA).

## 3. Results

**Figure 1:**
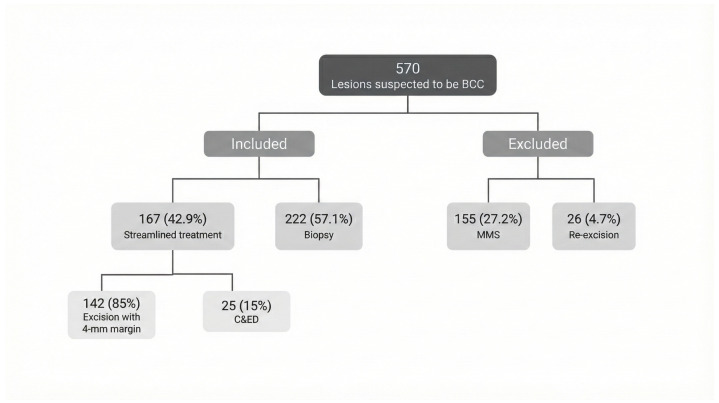
Flow diagram showing the proportion of suspected BCC lesion groups that were included or excluded from the study analysis. MMS = Mohs micrographic surgery; C&ED = curettage and electrodesiccation.

We identified 570 cases in which BCC was suspected according to the histopathology reports. After excluding cases treated with MMS (27.2%; n = 155) and those representing re-excisions (4.7%; n = 26), 389 lesions (68.2%) met the inclusion criteria and were included in the analysis. Of these, 42.9% (n = 167) were lesions treated with streamlined management (85%; n = 142 with 4 mm margins and 15%; n = 25 via C&ED). The remaining 57.1% (n = 222) were lesions biopsied before definitive treatment (Figure 1).

### 3.1. Group Characteristics: Biopsy vs. Streamlined Management (Table 1)

There was a higher proportion of men in the streamlined group (67.1%; n = 112) compared to the biopsy group (48.2%; n = 107) (*p* < 0.001). The mean age was higher in the streamlined group (68.5 ± 13.9 vs. 65.9 ± 12.6; *p* = 0.03).

The tumor location was significantly associated with the management (*p* < 0.001). Preoperative biopsy was more common for lesions in the H zone (59.5%; n = 132), representing 76.3% of procedures in this zone, while streamlined management was predominant in the L zone (54.5%; n = 91), representing 66.4% of procedures in this location. This association was also significant when analyzed by specific anatomical location (*p* < 0.001). Preoperative biopsies were more frequent for the head area (69.8%; n = 155) than in the streamlined group (35.3%; n = 59; *p* < 0.001). Streamlined management was also more common for lesions located on the back (22.8%; n = 38 vs. 5%; n = 11 in the biopsy group; *p* < 0.001) and upper extremities (16.2%; n = 27 vs. 5.4%; n = 12 in the biopsy group; *p* < 0.001).

Histopathological subtype analysis revealed a significant association with management type, with a higher proportion of aggressive BCC subtypes in the biopsy group (18%; n = 40) compared with the streamlined group (11.4%; n = 19) (*p* = 0.001).

**Table 1:** Clinical and demographic characteristics of basal cell carcinomas treated with streamlined management vs. preoperative biopsy approach.

Characteristic	Streamlined Group, n (%)	Biopsy Group, n (%)	*p*-Value
**Total (n)**	167	222	
Age (mean, SD)	68.51 ± 13.98	65.9 ± 12.59	0.03
Sex (Male)	112 (67.1)	107 (48.2)	<0.001
Anatomic zone (H, M, L)			<0.001
H	41 (24.6)	132 (59.5)	
M	35 (21)	44 (19.8)	
L	91 (54.5)	46 (20.7)	
Anatomic location			<0.001
Head	59 (35.3)	155 (69.8)	
Neck	6 (3.6)	1 (0.5)	
Thorax	9 (5.4)	10 (4.5)	
Abdomen	7 (4.2)	4 (1.8)	
Back	38 (22.8)	11 (5)	
Anogenital	0	1 (0.5)	
Upper limbs	27 (16.2)	12 (5.4)	
Lower limbs	21 (12.6)	28 (12.6)	
Subtype			0.03
Infiltrative	2 (1.2)	2 (0.9)	
Micronodular	4 (2.4)	7 (3.2)	
Morpheaform	1 (0.6)	11 (5)	
Nodular	73 (43.7)	64 (28.8)	
Superficial	58 (34.7)	40 (18)	
Mixed	20 (12)	21 (9.5)	
Other (no BCC)	9 (5.4)	77 (34.7)	
Aggressive histopathological subtype *	19 (11.4)	40 (18)	0.001
Recurrent BCC	7 (4.2)	5 (2.3)	0.290
Perineural invasion	0	0	NA
Radiotherapy **	8 (4.8)	7 (3.2)	0.413
Immunosuppression	3 (1.8)	7 (3.2)	0.399

Abbreviations: BCC = basal cell carcinoma; SD = standard deviation; H = high risk; M = medium risk; L = low risk; NA = not available. * Mixed BCCs with any aggressive component were also considered to have an aggressive histopathological subtype. ** Radiotherapy in the same area but for a different diagnosis, not for BCC.

#### 3.1.1. Multivariate Analysis Streamlined Management vs. Biopsy

To further assess independent predictors of management strategy, a multivariable logistic regression model was performed. Increasing patient age was significantly associated with greater odds of receiving streamlined management (OR = 1.03; 95% CI: 1.01–1.05; *p* = 0.011. Anatomical location was a strong and significant predictor: lesions in high-risk areas had a lower probability of streamlined management (H zone: OR 0.14; *p* < 0.001; M zone: OR 0.45; *p* = 0.026). The presence of an aggressive histopathological subtype was also negatively associated with streamlined management (OR = 0.48; 95% CI: 0.25–0.95; *p* = 0.035). Male sex showed a non-significant trend toward streamlined treatment (OR = 1.59; *p* = 0.083).

#### 3.1.2. Streamlined Group Sub Analysis: 4 mm Margins vs. C&ED

When analyzing subgroups of patients treated in the streamlined management group (4 mm excision vs. C&ED) a higher proportion of men was observed in the C&ED group (92%; n = 23 vs. 62.7%; n = 89; *p* = 0.004). In the subgroup of lesions excised with 4 mm margins, there was a significantly higher proportion of lesions located on the head compared with the group treated with C&ED (40.8%; n = 58 vs. 4%; n = 1; *p* = 0.001). In the C&ED group, the lesions were predominantly located in M and L anatomical zones (96%; n = 24). The most common histopathological subtype in the C&ED group was the superficial subtype (64%; n = 16), while in the 4 mm excision group, it was the nodular subtype (47.9%; n = 68) (*p* = 0.008). No significant differences were observed regarding the excision of BCCs with aggressive histopathological subtypes between the two groups (12%; n = 17 vs. 8%; n = 2 in the excision and C&ED groups, respectively; *p* = 0.59) (Table 2).

#### 3.1.3. Multivariate Analysis Streamlined Subgroup

In the multivariable analysis, male sex was significantly associated with a higher likelihood of receiving treatment with C&ED (OR 17.8; 95% CI 2.14–148.4; *p* = 0.008), indicating a strong influence of sex on surgical management choice. In contrast, neither the aggressive histologic subtype (OR 0.74; 95% CI 0.15–3.76; *p* = 0.715) nor age (OR 0.98 per year; 95% CI 0.94–1.02; *p* = 0.236) were significantly associated with treatment type. Anatomical location also did not reach overall statistical significance (*p* = 0.160), although there was a borderline trend toward lower likelihood of C&ED for tumors in high-risk zones (H zone: OR 0.13; 95% CI 0.02–1.07; *p* = 0.058).

### 3.2. Outcome Comparison

**Figure 2:**
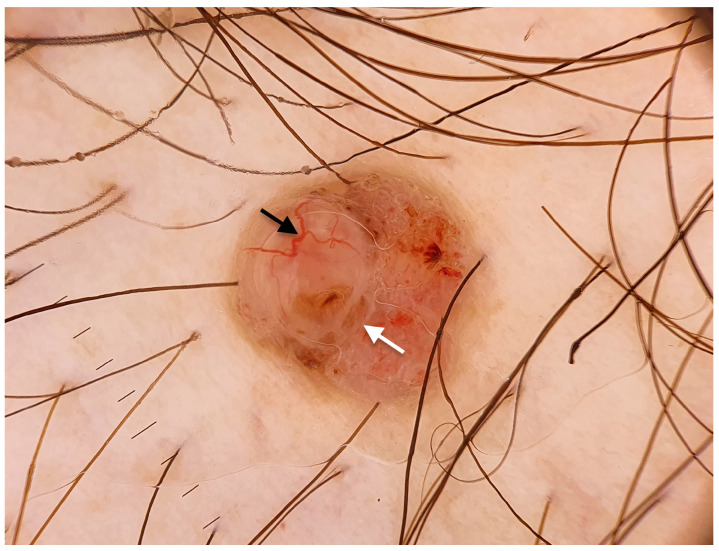
Dermoscopic image of a poroid hidradenoma mimicking a basal cell carcinoma. The lesion showed arborizing vessels (black arrow) and globules (white arrow) (polarized light dermoscopy, original magnification 10×).

In the streamlined group, 94.6% (n = 158) of cases were confirmed as BCC, compared with 73.4% (n = 163) in the biopsied lesions group (*p* < 0.001) (Figure 2). Consequently, presumptive diagnostic errors were significantly lower in the streamlined group (5.4% vs. 26.6%; *p* < 0.001). After adjusting for age, sex, and anatomical site, cases in the streamlined management group had significantly lower odds of presumptive diagnostic error compared with the biopsied group (adjusted OR = 0.13, 95% CI: 0.059–0.285; *p* < 0.001). Age, sex, and anatomical location (H-, M-, or L-risk zones) were not significantly associated with diagnostic accuracy (all *p* > 0.2). In both groups, the most common presumptive diagnostic error (lesions clinically suspected and managed as BCC but later found to have a different diagnosis) was squamous cell carcinoma (SCC) (33.3%; n = 3 in the streamlined group vs. 20.3%; n = 12 in the biopsy group), followed by actinic keratosis in the biopsy group (18.6%; n = 11) and adnexal tumors (11.1%; n = 1 in the streamlined group vs. 11.9%; n = 7 in the biopsy group). All SCCs were low-risk (1 well-differentiated and 2 SCC in situ) (Table 3).

For streamlined BCCs treated with direct excisions with 4 mm margins, negative margins were achieved in 97.9% (139/142) of the cases. Histopathological margin involvement was significantly associated with the presence of any high-risk factor (4.8%; n = 3 vs. 0%; n = 0; *p* = 0.048) and, with recurrent BCC (i.e., previously treated) among cases managed with streamlined treatment (33.3%; n = 1 vs. 4.4%; n = 6; *p* = 0.023). The three cases with positive margins were located in the H zone but had non-aggressive histopathological subtypes, including two nodular and one superficial BCC. Margin involvement was not significantly associated with high-risk anatomical locations (*p* = 0.127), although 100% (n = 3) of cases with positive margins were located in the H zone.

Among lesions suspected to be BCC that underwent streamlined treatment, 24.6% (n = 41) were located in the H zone. This group had a significantly higher mean age compared with those that had lesions in other areas (72.3 ± 12.8 years vs. 67.4 ± 14 years; *p* = 0.048). No significant associations were found with sex (*p* = 0.34) or histopathological subtype (*p* = 0.42). Additionally, there were no significant differences in diagnostic accuracy (*p* = 0.53) or margin involvement (*p* = 0.13) within this group.

### 3.3. Discussion

Our findings demonstrate that streamlined management through direct excision with predefined clinical margins or C&ED is an effective strategy in patients with lesions highly suggestive of BCC by clinical and dermoscopic findings, showing minimal diagnostic error and a low proportion of positive margins among excised lesions. Numerous highly accurate diagnostic techniques for BCC exist nowadays, with dermoscopy being the most widely used due to its cost-effectiveness as well as its high sensitivity and specificity [3]. This tool can even predict histopathological subtypes with a notable degree of accuracy [9,10]. Interestingly, the 2023 European guidelines for the diagnosis and treatment of BCC recommend, with a grade C endorsement and unanimous agreement, that superficial and nodular BCCs can be diagnosed using clinical examination supported by non-invasive techniques without the need for histopathological confirmation [11]. This enables the careful selection of low-risk BCCs for direct excision with predefined margins or destructive treatment without requiring a prior biopsy.

In our study, dermoscopy demonstrated a diagnostic accuracy of nearly 95% for lesions suspected to be BCC in the streamlined group, significantly outperforming the accuracy observed in the biopsy group (73%; *p* < 0.001). Although this difference seems large, it is likely due to selection bias, as preoperative biopsies are typically performed on lesions with ambiguous clinical and dermoscopic features, where histopathological confirmation is considered necessary. Therefore, it is crucial for clinicians to have a high level of diagnostic certainty when opting for streamlined management, ensuring that only appropriately selected lesions are managed without biopsy. Moreover, since we had a 5% error rate, submitting all material for postoperative histopathological evaluation is recommended when performing streamlined management. Newer diagnostic tools, such as RCM and hyperspectral imaging [12], have shown even greater diagnostic precision [13], with accuracy comparable to traditional histopathological methods for BCC classification [14]. Therefore, we believe that incorporating these non-invasive diagnostic modalities could further enhance bedside BCC diagnostic accuracy.

In our study, 97.9% of tumors excised with 4 mm margins without prior biopsy achieved negative margins, aligning with existing literature showing that 4 mm margins can achieve a complete excision rate > 95% [15]. Defining these margins with dermoscopy is key, as it outperforms the naked eye in delineating tumor margins [15]. Notably, excisions of high-risk BCC were significantly associated with margin involvement, with recurrent BCC being the only risk factor significantly associated with positive margins. This can be attributed to the greater subclinical spread of recurrent BCCs, classifying them as high-risk tumors that often necessitate wider margins for complete excision [16,17]. For cases where MMS is not an option despite the presence of high-risk factors (i.e., recurrence, size, or aggressive histopathological subtypes), some guidelines recommend excision with clinical margins > 5 mm, compared to the 3 to 4 mm margins proposed for low-risk BCCs [11,14,18,19].

MMS remains the preferred treatment for BCC arising on high-risk areas due to its tissue-sparing properties and lower recurrence rates [20,21]. In our study, while biopsies were often prioritized for tumors in the H zone or suspected to be high-risk BCCs, streamlined management was still chosen in approximately 25% of cases without negatively impacting diagnostic accuracy or margin involvement when compared to the same outcomes in other anatomical areas. This approach was more frequently used in older patients, potentially due to greater skin elasticity that allows for primary closure of larger defects, lower concern about facial scarring, limited mobility and multi-day travel allowance, and a higher prevalence of comorbidities complicating more complex or repeated procedures [22]. Recent research highlights that older patients are less concerned with cosmetic outcomes and more focused on reducing the time between diagnosis and treatment or avoiding complex procedures, supporting a more individualized approach [23]. We propose that while MMS should remain the standard in high-risk areas, streamlined management with predefined clinical margins and postoperative margin assessment could be an appropriate option for small, well-defined tumors for which high diagnostic certainty is expected. This is especially relevant when primary closure is feasible and no complex tissue rearrangement (i.e., flaps) are predicted. This streamlined approach should be discussed with the patient, considering the associated risks and benefits. Since clinical examination and dermoscopy can provide the clinician with clues about BCCs being high- or low-risk subtypes [10], we otherwise mostly prefer limiting streamlined management to low-risk subtypes. In our practice, we tend to manage low-risk BCCs with streamlined management and high-risk BCCs with a biopsy and subsequent MMS.

Adopting a streamlined or “see and treat” strategy offers several advantages. Studies have shown that this approach significantly reduces costs and waiting times without compromising the quality of care, and it is often preferred by patients [7,24,25]. The streamlined strategy also reduces travel burdens, particularly for elderly patients, and facilitates treatment while lesions remain small, addressing concerns that delays could lead to tumor growth [5,7,23]. Although our study did not include follow-up data for recurrence detection, prior research by Wu et al. [5] suggests that recurrence rates with this approach are comparable to those of traditional management, with significant cost savings and without compromising clinical outcomes. Their findings were based on lesions < 1 cm in diameter and located on the trunk and extremities, which are considered low-risk areas [5]. The authors also performed a cost-effectiveness analysis demonstrating a combined weighted average savings per case of 95 USD (15% of total average cost) [5]. Given the worldwide lack of resources after the COVID-19 pandemic and already collapsed healthcare systems, with long waiting times, this streamlined alternative emerges as a relevant option [7,26]. Finally, we always recommend obtaining tissue for postoperative histopathological evaluation when performing streamlined management.

#### Limitations

Our study was based on the individual doctors’ judgment, a relatively limited sample size, and the absence of universally endorsed management criteria. Although no institutional protocol was in place, dermatologists selected lesions for streamlined treatment based on standardized and widely accepted clinical and dermoscopic features of BCC. This was a single-center study and the dermatologists working at the center have >10 years of experience using dermoscopy and are experienced surgeons. It should be noted that the streamlined approach requires substantial clinical and dermoscopic expertise, and its safety and effectiveness may depend on the experience and training of the treating clinicians. Therefore, the reported diagnostic accuracy and treatment outcomes might not be extrapolated to other centers.

Additionally, due to the retrospective nature of the study, each lesion was evaluated by a single dermatologist and the same lesions were not assessed independently by multiple observers; thus, formal interobserver reliability could not be assessed.

Importantly, data was not recorded on the dermoscopic high-risk features, tumor size, and the presence of well- vs. ill-defined margins. Thus, their influence on the chosen management (i.e., streamlined vs. biopsy) could not be evaluated. Additionally, the current analysis aggregates low-risk and high-risk BCCs in some of the results, which may lead readers to overestimate the safety of the streamlined excision approach for all tumor types. The individual dermatologists’ accuracy was not recorded and therefore the impact of different dermatologists in the analysis was not calculated. We also lack patient follow-up to compare recurrence rates. A prospective study would be ideal for evaluating both treatment modalities.

## 4. Conclusions

Our study demonstrates that streamlined management, without the need for a preoperative biopsy, is a highly effective, and patient-convenient alternative for the management of BCC. This is especially relevant for low-risk cases in older patients who either prefer less complex treatments or wish to avoid delays in definitive management, and in settings with limited resources or long waiting times. By reducing the need for multiple visits, this approach also allows for faster treatment initiation. To further validate our findings, future prospective studies with larger sample sizes from multiple centers and longer follow-up periods are necessary. The emergence of novel non-invasive in vivo and ex vivo techniques could also serve in this streamlined management [27]. Additionally, refining the selection criteria for direct excision could optimize outcomes and expand its applicability to a broader range of BCC cases. In the future, the streamlined approach could also be integrated into the monitoring of therapeutic response to systemic treatments, potentially replacing the need for incisional biopsies by incorporating non-invasive imaging technologies such as RCM and OCT, as suggested by recent reports in the literature [28,29,30].

## Figures and Tables

**Table 2:** Subgroup analysis of lesions managed with streamlined treatment: direct excision with 4 mm margins vs. curettage and electrodessication.

Characteristic	Excision with 4 mm Margins, n (%)	C&ED, n (%)	*p*-Value
**Total (n)**	142 (85)	25 (15)	
Age (mean, SD)	68.91 ± 14.6	66.28 ± 9.85	0.388
Sex (Male)	89 (62.7)	23 (92)	0.004
Anatomic zone (H, M, L)			0.033
H	40 (28.2)	1 (4)	
M	29 (20.4)	6 (24)	
L	73 (51.4)	18 (72)	
Anatomic location			0.001
Head	58 (40.8)	1 (4)	
Neck	6 (4.2)	0	
Thorax	5 (3.5)	4 (16)	
Abdomen	7 (4.9)	0	
Back	30 (21.1)	8 (32)	
Anogenital	0	0	
Upper limbs	21 (14.8)	6 (24)	
Lower limbs	15 (10.6)	6 (24)	
Subtype			0.008
Infiltrative	1 (0.7)	1 (4)	
Micronodular	5 (3.5)	0	
Morpheaform	1 (0.7)	0	
Nodular	68 (47.9)	5 (20)	
Superficial	42 (29.6)	16 (64)	
Mixed	17 (12)	1 (4)	
Other (no BCC)	7 (4.9)	2 (8)	
Aggressive histopathologic subtype	17 (12)	2 (8)	0.588
Recurrent BCC	7 (4.9)	0	0.309
Perineural invasion	0	0	NA
Radiotherapy **	8 (5.6)	0	0.609
Immunosuppression	3 (2.1)	0	0.607

Abbreviations: BCC = basal cell carcinoma; SD = standard deviation; H = high risk; M = medium risk; L = low risk; NA = not available ** Radiotherapy in the same area but for a different diagnosis, not for BCC.

**Table 3:** Final histopathological diagnosis of suspected basal cell carcinomas managed with streamlined management vs. preoperative biopsy approach.

Characteristic	Streamlined Group	Biopsy Group	*p*-Value
	n = 167 (n; %)	n = 222 (n; %)	
Negative margins	139 (97.9) *	NA	
**Diagnostic error**	**9 (5.4)**	**59 (26.6)**	**<0.0001**
SCC	3 (33.3)	12 (20.3)	
AK	0	11 (18.6)	
Adnexal Tumor	1 (11.1)	7 (11.9)	
Folliculitis	0	6 (10.2)	
Seborrheic Keratosis	1 (11.1)	5 (8.4)	
LPLK	0	4 (6.8)	
Dermal nevus	1 (11.1)	4 (6.8)	
Unspecific/Normal skin	0	3 (5.1)	
Sebaceous Hyperplasia	1 (11.1)	2 (3.4)	
Telangiectatic Granuloma	0	2 (3.4)	
Cyst	0	1 (1.7)	
Melanoma	0	1 (1.7)	
Foreign body	0	1 (1.7)	
Atypical nevus	1 (11.1)	0	
Sebaceous nevus	1 (11.1)	0	

Abbreviations: SCC: Squamous Cell Carcinoma; AK: Actinic Keratosis; LPLK: Lichen Planus Like Keratosis. * Percentage calculated using as the denominator the total number of lesions excised with 4 mm margins without prior biopsy (n = 142).

## Data Availability

The data that support the findings of this study are available from the corresponding author upon reasonable request.

## References

[1] Schmults C.D., Blitzblau R., Aasi S.Z., Alam M., Amini A., Bibee K., Bordeaux J., Chen P.L., Contreras C.M., DiMaio D. (2023). Basal Cell Skin Cancer, Version 2.2024, NCCN Clinical Practice Guidelines in Oncology. J. Natl. Compr. Cancer Netw..

[2] Kim J.Y.S., Kozlow J.H., Mittal B., Moyer J., Olencki T., Rodgers P., Work Group, Invited Reviewers (2018). Guidelines of care for the management of basal cell carcinoma. J. Am. Acad. Dermatol..

[3] Reiter O., Mimouni I., Gdalevich M., Marghoob A.A., Levi A., Hodak E., Leshem Y.A. (2019). The diagnostic accuracy of dermoscopy for basal cell carcinoma: A systematic review and meta-analysis. J. Am. Acad. Dermatol..

[4] Lupu M., Popa I.M., Voiculescu V.M., Caruntu A., Caruntu C. (2019). A Systematic Review and Meta-Analysis of the Accuracy of in Vivo Reflectance Confocal Microscopy for the Diagnosis of Primary Basal Cell Carcinoma. J. Clin. Med..

[5] Wu X., Elkin E.B., Jason Chen C.S., Marghoob A. (2015). Traditional versus streamlined management of basal cell carcinoma (BCC): A cost analysis. J. Am. Acad. Dermatol..

[6] Abramson A.K., Krasny M.J., Goldman G.D. (2013). Tangential shave removal of basal cell carcinoma. Dermatol. Surg..

[7] McLaughlin S.J.P., Kenealy J., Locke M.B. (2018). Effect of a See and Treat clinic on skin cancer treatment time. ANZ J. Surg..

[8] Cameron M.C., Lee E., Hibler B.P., Giordano C.N., Barker C.A., Mori S., Cordova M., Nehal K.S., Rossi A.M. (2019). Basal cell carcinoma: Contemporary approaches to diagnosis, treatment, and prevention. J. Am. Acad. Dermatol..

[9] Reiter O., Mimouni I., Dusza S., Halpern A.C., Leshem Y.A., Marghoob A.A. (2021). Dermoscopic features of basal cell carcinoma and its subtypes: A systematic review. J. Am. Acad. Dermatol..

[10] Ceder H., Backman E., Marghoob A., Navarrete-Dechent C., Polesie S., Reiter O., Paoli J. (2024). Importance of Both Clinical and Dermoscopic Findings in Predicting High-Risk Histopathological Subtype in Facial Basal Cell Carcinomas. Dermatol. Pract. Concept..

[11] Peris K., Fargnoli M.C., Kaufmann R., Arenberger P., Bastholt L., Seguin N.B., Bataille V., Brochez L., Del Marmol V., Dummer R. (2023). European consensus-based interdisciplinary guideline for diagnosis and treatment of basal cell carcinoma—Update 2023. Eur. J. Cancer.

[12] Lin T.-L., Mukundan A., Karmakar R., Avala P., Chang W.-Y., Wang H.-C. (2025). Hyperspectral Imaging for Enhanced Skin Cancer Classification Using Machine Learning. Bioengineering.

[13] Fünfer K., Mozaffari M., Mayer O., Schlingmann S., Welzel J., Schuh S. (2024). One-Stop Shop: Diagnosis and Treatment of Basal Cell Carcinoma in One Step. J. Clin. Med..

[14] Kadouch D.J., Leeflang M.M., Elshot Y.S., Longo C., Ulrich M., van der Wal A.C., Wolkerstorfer A., Bekkenk M.W., de Rie M.A. (2017). Diagnostic accuracy of confocal microscopy imaging vs. punch biopsy for diagnosing and subtyping basal cell carcinoma. J. Eur. Acad. Dermatol. Venereol..

[15] Chen W., Liu Z.R., Zhou Y., Liu M.X., Wang X.Q., Wang D.G. (2022). The effect of dermoscopy in assisting on defining surgical margins of basal cell carcinoma. Dermatol. Ther..

[16] Hoorens I., Batteauw A., Van Maele G., Lapiere K., Boone B., Ongenae K. (2016). Mohs micrographic surgery for basal cell carcinoma: Evaluation of the indication criteria and predictive factors for extensive subclinical spread. Br. J. Dermatol..

[17] Mosterd K., Krekels G.A., Nieman F.H., Ostertag J.U., Essers B.A., Dirksen C.D., Steijlen P.M., Vermeulen A., Neumann H.A.M., Kelleners-Smeets N.W. (2008). Surgical excision versus Mohs’ micrographic surgery for primary and recurrent basal-cell carcinoma of the face: A prospective randomised controlled trial with 5-years’ follow-up. Lancet Oncol..

[18] Nasr I., McGrath E.J., Harwood C.A., Botting J., Buckley P., Budny P.G., Fairbrother P., Fife K., Gupta G., Hashme M. (2021). British Association of Dermatologists’ Clinical Standards Unit. British Association of Dermatologists guidelines for the management of adults with basal cell carcinoma 2021. Br. J. Dermatol..

[19] Lang B.M., Balermpas P., Bauer A., Blum A., Brölsch G.F., Dirschka T., Follmann M., Frank J., Frerich B., Fritz K. (2019). S2k Guidelines for Cutaneous Basal Cell Carcinoma - Part 2: Treatment, Prevention and Follow-up. J. Dtsch. Dermatol. Ges..

[20] Brown A.C., Brindley L., Hunt W.T.N., Earp E.M., Veitch D., Mortimer N.J., Salmon P.J., Wernham A. (2022). A review of the evidence for Mohs micrographic surgery. Part 2: Basal cell carcinoma. Clin. Exp. Dermatol..

[21] Van Loo E., Mosterd K., Krekels G.A., Roozeboom M.H., Ostertag J.U., Dirksen C.D., Steijlen P.M., Neumann H.M., Nelemans P.J., Kelleners-Smeets N.W. (2014). Surgical excision versus Mohs’ micrographic surgery for basal cell carcinoma of the face: A randomised clinical trial with 10 year follow-up. Eur. J. Cancer.

[22] Van Coile L., Verhaeghe E., Ongenae K., Destrooper L., Mohamadi Z., Brochez L., Hoorens I. (2023). The therapeutic dilemma of basal cell carcinoma in older adults: A review of the current literature. J. Geriatr. Oncol..

[23] Van Coile L., Meertens A., Shen A., Waalboer-Spuij R., Vossaert K., Verhaeghe E., Brochez L., Hoorens I. (2024). The impact of basal cell carcinoma on the quality-of-life in older patients. Sci. Rep..

[24] Javaid M., Imran D., Moncrieff M., O’Neill T.J., Sassoon E.M. (2004). The see-and-treat clinic in plastic surgery: An efficient, cost-effective, and training-friendly setup. Plast. Reconstr. Surg..

[25] Nightingale J., Travers L., Campbell J., Huang J., Green M., Warren T., Fitzgerald G. (2021). Outpatient surgical management of non-melanoma skin cancers of the head and neck in a regional center: An analysis of costs and outcomes. ANZ J. Surg..

[26] Gorman M., Coelho J., Gujral S., McKay A. (2015). One-Stop Clinic Utilization in Plastic Surgery: Our Local Experience and the Results of a UK-Wide National Survey. Plast. Surg. Int..

[27] Navarrete-Dechent C., Rajadhyaksha M., Nehal K.S. (2020). Perioperative Noninvasive Optical Imaging: A Changing Paradigm for Management of Keratinocyte Carcinomas. J. Investig. Dermatol..

[28] Villani A., Fabbrocini G., Costa C., Scalvenzi M. (2021). Reflectance Confocal Microscopy Identification of Subclinical Basal Cell Carcinoma after Vismodegib Treatment: Report of a Case. Dermatol. Ther..

[29] Cappilli S., Mannino M., Palmisano G., Bocchino E., Piccerillo A., Paradisi A., Di Stefani A., Peris K. (2025). Locally advanced basal cell carcinoma treated with sonidegib: In vivo monitoring with line-field confocal optical coherence tomography. Ski. Health Dis..

[30] Venturi F., Rapparini L., Sgarzani R., Scotti B., Campione E., Dika E. (2025). Cytoreductive approach with hedgehog inhibitors followed by reflectance confocal microscopy assisted Mohs surgery for morpheiform basal cell carcinoma. JEADV.

